# Clinical and mechanistic insights into the expression of SP100 family proteins in various cancers: a systematic review

**DOI:** 10.1186/s12885-025-15159-9

**Published:** 2025-11-04

**Authors:** Mehrnoosh Azami, Fatemeh Sadeghi, Nazanin Mohammadi, Zahra Sadat Shayegh, Arshia Hassanzadeh, Mohammad Javad Heidarzadeh, Armin ZarinKhat, Zhina Mohamadi

**Affiliations:** 1 Faculty of Medicine, Iran University of Medical Sciences, Tehran, Iran; 2 Tehran branch, Faculty of Medicine, Islamic Azad University of Medical Sciences, Tehran, Iran; 3 Faculty of Medicine, Tehran University of Medical Sciences, Tehran, Iran; 4 Islamic Azad University of Tehran- Medical Branch, Tehran, Iran; 5 Faculty of Medicine, Ahvaz University of Medical Sciences, Ahvaz, Khouzestan Iran; 6 Faculty of medicine, Kermanshah University of medical sciences, Shahid beheshti Blvd, Kermanshah, 6715847141 Iran

**Keywords:** Cancer, Neoplasm, SP100, SP140, SP110, SP140L

## Abstract

**Introduction:**

Cancer remains a leading global health burden, with projections estimating 35 million new cases by 2050. The Speckled Protein 100 (SP100) family, comprising SP100, SP110, SP140, and SP140L, has emerged as a critical player in cancer biology due to their roles in transcriptional regulation, chromatin modification, and immune signaling. While SP100 acts as a tumor suppressor in malignancies like breast cancer, SP110 and SP140 are implicated in promoting oral squamous cell carcinoma and glioma progression, respectively, and SP140L is linked to poor prognosis in pancreatic adenocarcinoma. Despite growing evidence of their dual oncogenic and tumor-suppressive functions across cancers, inconsistencies in expression patterns, regulatory mechanisms, and clinical implications persist. The current review of the SP100 family’s roles in cancer aims to clarify any direct future translational research to enhance cancer outcomes.

**Method:**

Following PRISMA guidance, a comprehensive search was conducted in several databases (PubMed, Scopus, Web of Science, and Google Scholar). After RAYYAN-assisted deduplication and screening, studies analyzing SP100, SP110, SP140, or SP140L alterations in human cancers (excluding cell lines studies) were included. Data on genetic/epigenetic changes, cancer types, and clinical outcomes were extracted, with quality assessed via standardized tools.

**Results:**

This systematic review analyzed 29 studies (2004–2024) to evaluate the dual roles of SP100 family proteins (SP100, SP110, SP140, SP140L) across 25 cancer types. SP100 exhibited context-dependent roles: high expression correlated with better prognosis in breast/lung cancers but poorer outcomes in glioma/PAAD, while low expression in LSCC was linked to tumorigenesis. SP110 overexpression was tied to poor prognosis in oral, lung, and renal cancers but was protective in DLBCL. SP140, the most studied member, showed divergent associations: high expression worsened outcomes in ccRCC, glioma, and gynecologic cancers but improved survival in AML, osteosarcoma, and melanoma. Genetic/epigenetic alterations (mutations, copy number loss, and methylation) influenced prognosis in hematologic and solid tumors. SP140L, the least explored, correlated with poor PAAD prognosis.

**Conclusion:**

This study highlights the dual oncogenic/tumor-suppressive roles of SP100 family proteins across cancers. It also introduces this family as a therapeutic target. Further, it underscores their prognostic value in hematologic and solid tumors, highlighting SP140 as a key biomarker and SP100/SP140 inhibitors as promising strategies. Geographic bias and limited SP140L data reveal gaps; Future research should include multi-center studies, standardized methods, and mechanistic exploration of SP140L and SP110 in the immune microenvironment. Prioritizing clinical trials targeting SP100 family members could advance precision oncology and immunotherapy integration.

**Supplementary Information:**

The online version contains supplementary material available at 10.1186/s12885-025-15159-9.

## Introduction

Cancer is a leading cause of mortality worldwide, accounting for nearly one in six deaths overall (16.8%) and one in five deaths from noncommunicable diseases (NCDs) (22.8%). It is also a primary driver of premature mortality, responsible for three out of ten premature NCD deaths (30.3%) among adults aged 30 to 69. In 177 of 183 countries, cancer ranks among the top three causes of death for this age group. With incidence rates rising, new cancer cases are projected to reach 35 million annually by 2050 [1].

The Speckled Protein 100 (SP100) family includes four members: SP100, SP110, SP140, and SP140L. These proteins contain functional domains such as PHD (plant homeodomain), SAND (named after proteins that contain it: SP100, Aire, NucP41/P75, and DEAF), and bromodomains, which interact with DNA or post-translationally modified histones. By containing a caspase activation and recruitment domain (CARD), SP proteins facilitate multimerization [2]. High expression levels of SP family genes have been linked to enriched transcriptional profiles. As a result, these genes are associated with inflammatory responses and TNF-α signaling through NF-κB. Therefore, SP family proteins may serve as effective predictors for immunotherapeutic approaches, potentially enhancing treatment outcomes [3].

The SP100 family of chromatin “reader” proteins, located on chromosome 2q37.1, comprises four closely related members: SP100, SP110, SP140, and SP140L. These proteins are characterized by functional domains, including SAND, plant homeodomain (PHD), bromodomain (BRD), and a caspase activation and recruitment domain (CARD), that enable DNA binding, recognition of histone modifications, and protein multimerization. This domain architecture suggests a central role in transcriptional regulation and immune modulation. As interferon-inducible proteins, SP family members are highly expressed in innate and adaptive immune cells (with SP140 expression being immune-restricted) and localize to promyelocytic leukemia nuclear bodies (PML-NBs). These dynamic, membraneless organelles are key sites for the transcriptional repression of both host and viral genomes. The clinical significance of this family is underscored by mutations linked to various diseases, including Crohn’s disease, multiple sclerosis, chronic lymphocytic leukemia, and veno-occlusive disease with immunodeficiency [4–8]. High expression levels of SP family genes have been linked to enriched transcriptional profiles, associating these proteins with inflammatory responses and TNF-α signaling through the NF-κB pathway. Therefore, SP family proteins may serve as effective predictors for immunotherapeutic approaches, potentially enhancing treatment outcomes.

The members of this family are shown to have several roles in malignancies. SP100, the most extensively studied member, plays a crucial role in enhancing the expression of p53-regulated genes [9]. It has been observed at lower expression levels in malignant tissues compared to normal mucosa at both the transcriptional and translational levels [10]. The gene SP100 regulates histone acetylation levels, which is shown to be closely associated with breast cancer prognosis, particularly in the HER2-enriched and basal-like subtypes. This suggests that the expression levels of this gene can enhance the prediction of breast cancer outcomes [11]. Furthermore, exploring the role of SP100 as a tumor suppressor in human fibroblasts revealed its function in maintaining cellular senescence and preventing malignant transformation of fibroblasts [12].

Another member, SP110, is significantly overexpressed in oral squamous cell carcinoma (OSCC). Its expression level correlates with clinical parameters such as methylation status, tumor stage, gender, race, and age, suggesting its potential utility as both a diagnostic and prognostic biomarker for OSCC [13].

On the other hand, SP140, another member of this family, is elevated in glioma and is associated with disease progression [14]. Similarly, in clear cell renal cell carcinoma, high SP140 expression is linked to poorer overall survival, positioning it as a potential prognostic biomarker [15]. Further, SP140L member is likely related to the occurrence, development, and poor prognosis of pancreatic adenocarcinoma (PAAD) [16].

Despite the growing evidence highlighting the diverse roles of the SP100 family in various malignancies, there remains a comprehensive review regarding patterns of their expression, regulation, inconsistencies, and functional implications across different cancer types. Therefore, we conducted this systematic hybrid literature review to pave the way for more targeted research and clinical applications. By ‘systematic hybrid literature review,’ we refer to a structured approach that combines predefined search criteria and critical study appraisal with narrative integration of expression patterns, functional roles, and mechanistic evidence.

## Method

This systematic review was planned and reported following the PRISMA 2020 (Preferred Reporting Items for Systematic Reviews and Meta-Analyses) guidance. PRISMA’s comprehensive checklist enhances systematic review planning and coordination [17]. Our systematic review has been registered on the Open Science Framework (OSF) (registration DOI 10.17605/OSF.IO/SKHF8) [18].

### Information sources and search strategy

A thorough search of four databases from the time of each database’s inception until October 5, 2024, was carried out. The databases included PubMed/MEDLINE, Scopus, Google Scholar and Web Of Science. As seen in Table 1, a controlled vocabulary that was enhanced with terms from each database was used to accomplish the objectives of this issue.

**Table 1:** Search strategy

DATABASE	SEARCH STRATEGY	RESULT
PUBMED/MEDLINE	(((((SP100[Title/Abstract]) OR (SP110[Title/Abstract])) OR (SP140[Title/Abstract])) OR (SP140L[Title/Abstract])) OR (“speckled protein“[Title/Abstract])) OR (“speckled proteins“[Title/Abstract])) AND (((((((((((cancer[Title/Abstract]) OR (cancers[Title/Abstract])) OR (carcinoma[Title/Abstract])) OR (carcinomas[Title/Abstract])) OR (malignancy[Title/Abstract])) OR (malignant[Title/Abstract])) OR (malignancies[Title/Abstract])) OR (neoplasm[Title/Abstract])) OR (neoplasms[Title/Abstract])) OR (“Neoplasms“[Mesh])) OR (“Carcinoma“[Mesh]))	130
WEB OF SCIENCE	((((((TS=(SP100)) OR TS=(SP110)) OR TS=(SP140)) OR TS=(SP140L)) OR TS=(speckled protein)) OR TS=(Speckled proteins) AND ((((((((TS=(Cancer)) OR TS=(Cancers)) OR TS=(Neoplasms)) OR TS=(Neoplasm)) OR TS=(Carcinoma)) OR TS=(Carcinomas)) OR TS=(Malignant)) OR TS=(Malignancies)) OR TS=(Malignancy))	528
SCOPUS	(TITLE-ABS-KEY(“SP100”) OR TITLE-ABS-KEY(“SP110”) OR TITLE-ABS-KEY(“SP140L”) OR TITLE-ABS-KEY(“SP140”) OR TITLE-ABS-KEY(“speckled protein”) OR TITLE-ABS-KEY(“speckled proteins”)) AND (TITLE-ABS-KEY(“cancer”) OR TITLE-ABS-KEY(“cancers”) OR TITLE-ABS-KEY(“neoplasm”) OR TITLE-ABS-KEY(“neoplasms”) OR TITLE-ABS-KEY(“malignant”) OR TITLE-ABS-KEY(“malignancy”) OR TITLE-ABS-KEY(“malignancies”) OR TITLE-ABS-KEY(“carcinoma”) OR TITLE-ABS-KEY(“carcinomas”))	192
GOOGLE SCHOLAR	All in title: “Cancer” AND “SP100""SP110""SP140""SP140L""speckled protein""speckled proteins”	10
Total		860

### Data screening and eligibility criteria

RAYYAN.ai was used to facilitate the blinded and independent screening of abstracts by multiple reviewers, leveraging AI-assisted relevance suggestions [19]. Rayyan was used to assist independent abstract screening. Titles and abstracts from 860 articles retrieved from our search strategy were blindly and independently screened by four reviewers (M.A, A.H, F.S, MJ.H). RAYYAN AI was employed to eliminate the duplicated records. The conflicts were resolved by another independent reviewer (A.Zkh).

All of the original English-language studies that investigated alterations in at least one of the SP100 family genes (including SP100, SP110, SP140, and SP140L) or outcomes associated with these alterations in human cancer samples were included (Table 2). This study focused on alterations in the SP100 family in human cancer patients and excluded research relying solely on commercially available cancer cell lines. During the initial screening phase, the inclusion criteria were only examined at the level of the articles’ titles and abstracts. However, in the subsequent phase, we screened the full text of included articles. Included articles whose full text was not available were excluded from the review.

**Table 2:** Step by step checklist to include an article. Failure to Meet the criteria of each step led to exclusion

1. A Journal article
2. An English language article
3. A human malignancy
4. A human sample
5. Alterations of the SP100 family
6. Article full text available

### Data extraction and quality assessment

In the subsequent phase of screening, we used the included articles’ full text to extract the following data: Study title, Author, Year, Country, Type of study, Aim of study, Population/Sample, Cancer type, Type of cell line, SP100 family member, Epigenetic/genetic alterations, Techniques used for measurement, Method, Outcome, Conclusion, and Risk of bias.

To assess the quality of the included studies and risk of bias, three assessors (M.A, N.M, A.H) used different tools based on the study design. The Newcastle-Ottawa Scale was used to evaluate observational studies (e.g., cohort, case-control), focusing on selection, comparability, and outcome domains [20]. The QUIPS tool ensured rigorous appraisal of prognostic studies linking SP100 family alterations to clinical outcomes (e.g., survival, treatment response) [21]. For experimental or bioinformatics components, Cochrane’s risk of bias framework addressed methodological rigor (e.g., measurement validity, selective reporting) [22]. This multi-tool approach aligned with the study’s focus on human cancer samples and mechanistic insights.

## Results

### Study selection

**Fig. 1:**
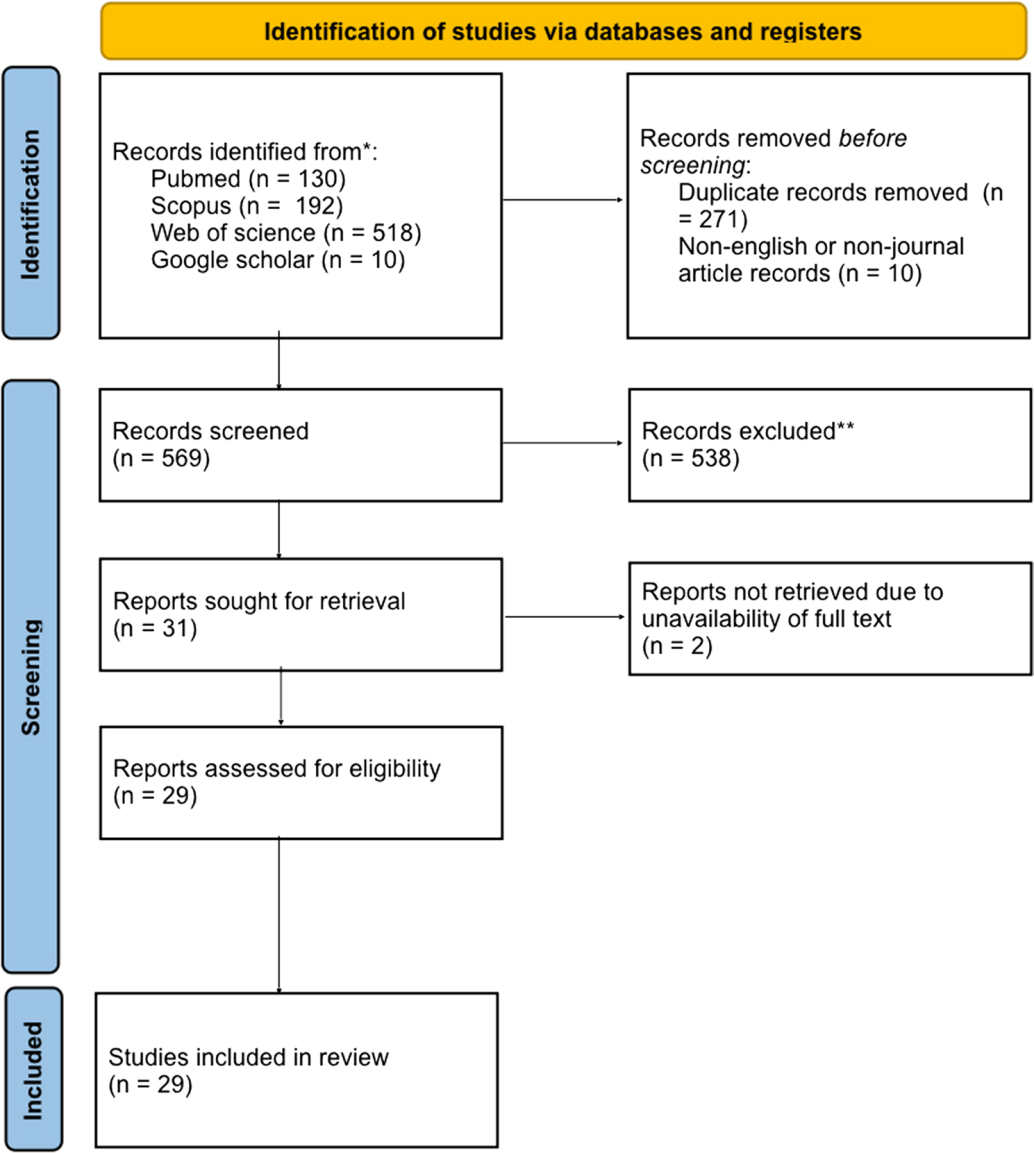
PRISMA 2020 flow diagram for new systematic reviews which included searches of databases

850 records were identified by our systematic search across the four aforementioned databases. 271 duplicates were excluded. We excluded 538 irrelevant studies by first screening the titles and then the abstracts of the remaining records. While the full text of 2 articles could not be retrieved. 10 non-English studies were excluded from the final selection due to language constraints. Eventually, we extracted data from 29 remaining articles (Fig. 1).

### Study characteristics

No explicit date restriction was applied during the search process; however, the retrieved studies were published between 2004 and 2024, reflecting the available literature identified by the search strategy. These studies were conducted in the following four countries: China with 22 studies (76%), the USA with 5 studies (17.2%), and Spain and South Korea each with 1 study (3.4%). In quality assessment, most of the studies showed low (51.7%) and moderate risk of bias (37.9%).

A total of 25 distinct cancer types were investigated in the retrieved studies that could be categorized in two groups:1. Hematologic malignancies, including Acute Myeloid Leukemia (AML), Chronic Myelomonocytic Leukemia (CMML), Mantle Cell Lymphoma (MCL), Diffuse Large B-Cell Lymphoma (DLBCL), Chronic Lymphocytic Leukemia (CLL), and Multiple Myeloma.2. Solid tumors like Clear Cell Renal Cell Carcinoma (ccRCC), Laryngeal Squamous Cell Carcinoma (LSCC), Colorectal Cancer (CRC), Pancreatic Adenocarcinoma (PAAD), Osteosarcoma, Glioma, Breast Cancer, Lung Cancer, Hepatocellular Carcinoma (HCC), Bladder Cancer (BLCA), Cervical Squamous Cell Carcinoma (CSCC), Ovarian Cancer, and Oral Squamous Cell Carcinoma (OSCC) (Table 3).

**Table 3:** Extracted data from the included studies

Study title	Author	Year	country	Type of study	Aim of study	Population/Sample	Cancer type	Type of cell line	SP100 family member	Epigenetic/genetic alterations	Techniques used for measurement	Method	Outcome	Conclusion	Risk of bias
Hypomethylation of TET2 Target Genes Identifies a Curable Subset of Acute Myeloid Leukemia [23]	Yamazaki J, et al.	2015	USA	cohort	Exploring the relationship between TET2 mutations, DNA methylation patterns, and clinical outcomes in patients with acute myeloid leukemia (AML).	94 AML patients for the test cohort and 92 AML patients for the validation cohort	AML	Myeloid line	SP140	Low methylation	bisulfite pyrosequencing	The study utilized bisulfite pyrosequencing to analyze the methylation status of four tet2-DMCs in 94 consecutive patients treated with cytarabine-based chemotherapy, using hierarchical clustering, Cox proportional hazards regression, and Kaplan Meier analyses.	The methylation status of four TET2-DMCs, including CpG sites near the SP140 transcription start site and in the gene bodies of MCCC1, EHMT1, and MTSS1, showed significant variability compared to normal peripheral blood and bone marrow.	Low methylation of SP100 is correlated with curable AML and better prognosis.	Moderate
Assessment for prognostic value of differentially expressed genes in immune microenvironment of clear cell renal cell carcinoma [24]	Yin X, et al.	2020	China	cohort	evaluating how specific genes, differentially expressed in the immune microenvironment of clear cell renal cell carcinoma (ccRCC), can serve as prognostic indicators	537 cases	Clear cell renal cell carcinoma	CCRC cells	SP140	High expression	Immunohistochemistry (IHC), flow cytometry (FCM) and next-gene sequencing (NGS)	collecting tumor samples, isolating RNA for gene expression profiling, and analyzing the data to identify genes with prognostic value	The study found that high immune scores in clear cell renal cell carcinoma were linked to poor overall survival, with significant differences in gene expression and immune infiltration, identifying key prognostic genes like sp140	High expression of SP140 is significantly associated with poor prognosis in clear renal cell carcinoma.	Low
Gene Signature Associated with Bromodomain Genes Predicts the Prognosis of Kidney Renal Clear Cell Carcinoma [15]	Lu J, et al.	2021	China	cohort	Investigating the expression levels of BRD genes in renal clear cell carcinoma	515 patients	Clear cell renal cell carcinoma	KIRC cells	sp100/sp110/sp140L/SP140	High expression of all	(1) Nomogram Analysis via R package (2) TIMER2 website (3) Immunohistochemistry marker	mRNA levels of tumor tissues and adjacent tissues were extracted from The Cancer Genome Atlas (TCGA) database. Seven BRD genes (*KAT2A*,* KAT2B*,* SP140*,* BRD9*,* BRPF3*,* SMARCA2*, and *EP300*) were searched by using LASSO Cox regression and the model with prognostic risk integration.	seven BRD genes can be utilized as new targets for KIRC.	SP100 family members are highly expressed in CRCC. SP140 can be utilized as new target for this cancer.	Low
Identification of tumor-associated proteins in well differentiated laryngeal squamous cell carcinoma by proteomics [25]	Zhou J, et al.	2007	China	observational	Identification of Tumor Markers	Eight human LSCC tissues were collected from eight surgically resected patients at Chong Qing Medical University’s First Affiliated Hospital, including eight men aged 57 years and above	laryngeal squamous cell carcinoma	squamous cell	SP140	High expression	2-DE & MS	used mass spectrometry and liquid chromatography to identify tumor-associated peptides	The study suggested that SP140 could be associated with immune responses in the tumor microenvironment, and its altered expression in LSCC may offer insight into the disease’s molecular mechanisms	sp140 upregulated	Low
Low expression of Sp100 in laryngeal cancer: Correlation with cell differentiation [10]	Wei Li, et al.	2010	China	observational	Exploring the expression of Sp100 and its potential clinical implications in laryngeal cancer, understanding the role of Sp100 in the initiation and progression of tumorigenesis	96 laryngeal cancer samples and paired normal epithelium	Laryngeal cancer	Laryngeal cancer cells	SP100	Downregulation	RT-PCR, Western blot and immuno-histochemical staining	Chi-squared test	Low Sp100 expression of both transcriptional and translational levels in the malignant tissues and downregulation among well-moderately and poorly-differentiated cancer cells.	Low expression of SP100 was detected in laryngeal cancer.	Moderate
Effects of TET2 mutations on DNA methylation in chronic myelomonocytic leukemia [26]	Yamazaki J, et al.	2012	USA	cohort	Investigating how mutations in the TET2 gene affect DNA methylation patterns in chronic myelomonocytic leukemia (CMML)	13 patients with CMML including 5 mutant and 8 wild-type	Chronic myelomonocytic leukemia	myelomonocytes	SP140	Hypermethylation	PCR & bisulfite pyrosequencing & mass spectromy	1: Sample Collection 2: Mutation analysis (pcr& bisulfite pyrosequencing) 3: Measurement of 5-methyl-cytosine levels by mass spectrometry. 4: Statistical analysis.	TET2 missense or nonsense mutations were detected in 53% of patients, with only 1/30 having IDH1 or IDH2 mutations. Two non-CpG island promoters, AIM2 and SP140, were hypermethylated in mutant TET2 patients.	DNA methylation levels of SP*140* are good markers for detecting *TET2* mutations, contributing to CMML.	Moderate
Genomic and transcriptomic profiling reveals distinct molecular subsets associated with outcomes in mantle cell lymphoma [27]	Yi S, et al.	2022	China	cohort	Identifying specific molecular subgroups within mantle cell lymphoma (MCL)	134 MCL patients	Mantle cell lymphoma		SP140	Mutations and Downregulation	Genomic Sequencing: WGS & WES Transcriptomic Profiling: RNA sequencing	1: Sample Collection 2: Genomic and Transcriptomic Analysis 3: Data Processing and Bioinformatics 4: Statistical Analysis	Our cohort found 8% SP140 mutations, with 9 out of 11 being frameshift and nonsense mutations causing truncated SP140. 7.5% had SP140 deletion, causing downregulation of expression.	Mutations in *SP140*, were predictive of poor progression-free survival.	Moderate
Uniparental disomies, homozygous deletions, amplifications, and target genes in mantle cell lymphoma revealed by integrative high-resolution whole-genome profiling [28]	Sílvia Bea, et al.	2009	Spain	observational	Identifying new genetic alterations and potential target genes relevant in the pathogenesis of MCL tumors	Mononuclear cells from the peripheral blood of 16 MCL patients,	Mantle cell lymphoma	10 MCL cell lines (HBL2, UPN1, MINO, REC1, GRANTA519, NCEB1, MAVER1, Z138, JEKO1, and JVM2) and 28 primary MCL	SP100	Polymorphism	PCR	FISH	Polymorphisms of SP100 were detected in MCL.	Polymorphism of SP100 may be potentially relevant in MCL pathogenesis.	Moderate
A CIC-related-epigenetic factors-based model associated with prediction, the tumor microenvironment and drug sensitivity in osteosarcoma [29]	Yu B, et al.	2024	China	observational	addressing several important aspects related to the role of CIC (Capicua) and epigenetic factors in osteosarcoma	148 patients	Osteosarcoma		SP140		RNA-seq_	The study used gene expression data to identify epigenetic factors linked to cancer immune phenotypes and built a prognostic risk model. It analyzed molecular subtypes, pathways, and tumor microenvironment, and developed a nomogram for survival prediction, also evaluating immune features, drug sensitivity, and immunotherapy responses	SFMBT2, SP140, CBX5, and HMGN2 were identified as protective factors, whereas SMARCA4, PSIP1, ACTR6, and CHD2 were identified as risk factors	SP140 was protective against OS.	Low
Stratifying osteosarcoma patients using an epigenetic modification-related prognostic signature: implications for immunotherapy and chemotherapy selection [30]	Li Zh, et al.	2024	China	Cohort	Developing an accurate prognostic signature for OS using epigenetic modification genes (EMGs).	85 OS patients’ data	Osteosarcoma	-	SP140	High expression		Expression levels and prognostic significance of SP family members were evaluated in the TCGA and CGGA datasets.	Patients with low SP140 expression (high-risk group) had significantly shorter survival.	Higher expression of SP140 is associated with better prognosis.	Moderate
Prognostic Value of a Stemness Index-Associated Signature in Primary Lower-Grade Glioma [31]	Zhang M, et al.	2020	China	observational	the aim was to develop a stemness index-based signature (SI-signature) for risk stratification and survival prediction	The high-throughput RNA-seq data of 529 patients with LGG from the TCGA database and 1,152 normal brain tissue samples	Glioma		SP100	High expression	Immunohistochemistry (IHC), ROC Curve Analysis RNA Sequencing (RNA-seq) Stemness Index Calculation (mRNAsi)	this study identified key DEGs in lower-grade glioma (LGG) using TCGA and GTEx data, analyzed their functions, and developed a stemness index (SI) signature associated with patient survival, which was validated with CGGA data.	Among the seven genes, ALOX5AP, APOBEC3C, GNG5, and SP100 were identified as risk-associated genes, whereas ADAP2, FCGRT, and LRRC25 were confirmed as protective genes.	SP100 was overexpressed in Glioma cells and was associated with poor outcome.	Low
SP140 inhibitor suppressing TRIM22 expression regulates glioma progress through PI3K/AKT signaling pathway [14]	Li X, et al.	2024	China	Cohort	Understanding clinical significance of SP100 family in glioma	Data from TCGA and CGGA datasets.	Glioma	Human astrocyte HA and human glioma cell lines U87, U118, and U251	sp100/sp110/sp140L/SP140	High expression plus deep deletions being the most common mutation type, followed by missense mutations for all family members.	CancerSEA and TIMER		High expression of SP family was correlated with a worse prognosis in glioma patients. SP140 is an independent prognostic factor.	SP140 can be a practical tool for predicting the survival of glioma patients, SP140 inhibitor could suppress glioma progress via TRIM22/PI3K/AKT signaling pathway. High expression of SP family was correlated with a worse prognosis in glioma patients.	Low
Genetic characteristics involving the PD-1/PD-L1/L2 and CD73/A2aR axes and the immunosuppressive microenvironment in DLBCL [32]	Zhang T, et al.	2022	China	Observational molecular genetics study	The study aims to investigate the genetic characteristics of the PD-1/PD-L1/L2 and CD73/A2aR pathways and assess their roles in the immunosuppressive tumor microenvironment in diffuse large B-cell lymphoma (DLBCL).	Tumor biopsies of 188 patients with DLBCL	Diffuse large B-cell lymphoma (DLBCL)		SP140	Translocation	Multiplex Immunofluorescence Staining and RNA Sequencing	1-Sequencing Techniques: Whole-Exome Sequencing/Targeted Deep Sequencing 2-RNA Sequencing 3-Single-Cell RNA Sequencing 4-Multiplex Immunofluorescence Staining	SP140: Identified as a novel translocation partner for PD-L1; inversion between PD-L1 and PD-L2 leads to increased PD-L1 expression.	• PD-L1 overexpression genetic basis. • Strategies inhibiting PD-1/PD-L1/L2, combined with CD73/A2aR, may be effective therapeutic options.	Moderate
Natural killer cell-associated prognosis model characterizes immune landscape and treatment efficacy of diffuse large B cell lymphoma [33]	Xiao, W, et al.	2024	China	Experimental study	Exploring and constructing a risk assessment model based on NK cell-related genes to accurately predict the prognosis and treatment efficacy in patients with Diffuse Large B-cell Lymphoma (DLBCL).	DLBCL patients from various databases, collected 244 NK cell-related genes, and identified 233 genes in DLBCL samples in the GEO dataset.	Diffuse large B cell lymphoma	B cell	SP110	Downregulation	PCR (qRT-PCR)	1-data collection 2-Identification of NK Cell-Related Genes 3-Risk Model Construction 4-Verification with qRT-PCR 5-Statistical Analysis	The study identified seven NK cell-related genes (MAP2K1, PRKCB, TNFRSF10B, IL18, LAMP1, RASGRP1, and SP110) that predict prognosis and treatment efficacy in DLBCL patients	the expression of sp110 decreased	low
The diagnostic significance of the ZNF gene family in pancreatic cancer: a bioinformatics and experimental study [34]	Zhu, L, et al.	2023	China	Bioinformatics and experimental	Investigating the diagnostic potential of the ZNF (zinc finger) gene family in pancreatic cancer	178 PAAD samples from the TCGA database and 171 normal samples	PAAD		SP110	High expression	qPCR	RNA-seq data for PAAD were sourced from TCGA and GEO databases, and DE-ZNFs were screened. An optimal risk model and prognostic value were established through Cox regression analyses.	Seven immune cells were found to be differentially expressed in high- and low-risk patients, with ZNF185, PRKCI, and RTP4 upregulated, while ZMAT1 and CXXC1 were downregulated in PAAD samples. Cell experiments confirmed the upregulation of RTP4, SERTAD2, and SP110.	Expression of SP110 is positively correlated with prognosis.	Moderate
Expression, prognostic value and mechanism of SP100 family in pancreatic adenocarcinoma (PAAD) [16]	Yunjie Duan, et al.	2023	China	Observational	Exploring more therapeutic targets and prognostic biomarkers to improve the prognosis of PAAD patients	cancerous and adjacent tissues of 92 patients with PAAD	PAAD	Pancreatic cancer cell lines and adjacent cells	sp100/sp110/sp140L/SP140	High expression	Analysis by R (version 3.6.3)	analysis by R (version 3.6.3)/Single-cell Analysis/Cytoscape, the STRING, GSCALite, Metascape, LinkedOmics, UALCAN, TIMER, cBioPortal, TCGA, TISIDB, and KaplanMeier Plotter databases	Mechanistically, TP53 mutations were significantly associated with the expression levels of the SP100 family members, which were significantly coexpressed with M6A methylation regulators and were activated in multiple oncogenic pathways, including the EMT pathways.	High expression of SP100 is significantly associated with poor prognosis in PAAD patients.	Low
Identify a DNA Damage Repair Gene Signature for Predicting Prognosis and Immunotherapy Response in Cervical Squamous Cell Carcinoma [35]	Hong Zh, et al.	2022	China	Observational and Bioinformatics Study	the study aims to uncover molecular signatures that could help predict patient prognosis and guide treatment decisions, particularly in the context of immunotherapy response in CESC	RNA-seq data of 306 CESC samples were obtained from TCGA database. Besides, samples with incomplete clinical information were excluded	Cervical and ovarian cancer		SP140	Loss	RNA seq_	1-data collection. 2-Identification of Differentially Expressed Genes. 3-Predictors of Immunotherapeutic Response 4-Validation of the Regulatory Mechanism of TFs	Thirty-five upstream TFs significantly associated with the DDR genes were identified by coexpression analysis, of which SP140 showed maximum copy number loss in CESC.There was clear correlation between the expression levels of SP140 and FBXO6.	FBXO6 Is Downregulated in CESC due to Loss of SP140 which is correlated with poor prognosis.	Moderate
SP100 expression modulates ETS1 transcriptional activity and inhibits cell invasion [36]	Yordy S, et al.	2004	USA	Experimental study	The study aims to investigate how SP100 expression modulates ETS1 transcriptional activity and its effects on cell invasion	HeLa cells and MDA-MB-231 breast cancer cells	Breast cancer		SP100	-	qRT-PCR and western Blotting Fluorescent tagging	The study used various experimental methods to explore the interaction between SP100 and ETS1 and its impact on cancer cell invasion. Through yeast two-hybrid screening, protein assays, and confocal microscopy, SP100 was identified as a binding partner of ETS1.	The study found that SP100 interacts with ETS1, repressing ETS1’s transcriptional activity on invasion-related genes and thereby reducing cancer cell invasiveness, with IFN-α further enhancing SP100’s inhibitory effect on ETS1 target gene expression	Expression of SP100 inhibits the invasion of breast cancer cells and is induced by Interferon-alpha, which has been shown to inhibit the invasion of cancer cells.	Moderate
A Histone Acetylation Modulator Gene Signature for Classification and Prognosis of Breast Cancer [11]	Long M, et al.	2021	China	In silico	Assessing regulators of histone acetylation to cure breast cancer.	1102 breast cancer patients’ data	Breast cancer	-	SP100	High expression	Nonnegative Matrix Factorization Clustering and Survival analyses through Kaplan–Meier	TCGA data sources were used for histone acetylation modulator analysis- 8 genes were found effective	High expression of SP100 in breast cancer was detected.	High expression of *SP100* was correlated with good prognosis.	Low
Bioinformatics and Prognosis Analysis of SP100 Gene in Lung Cancer Associated with Cerebral Infarction [37]	Jiacai L, et al.	2019	China	bioinformatics analysis	Exploring the correlation between the SP100 gene and lung cancer associated with cerebral infarction	2173 genes co-expressed with SP100 were screened from 18 cell expression data, containing 1688 up-regulated genes and 485 down-regulated genes	Lung cancer	Lung cancer cells	SP100	High expression	The lncRNA co-expression with mRNA is analyzed, and the genes with strong relationship were evaluated by GO and KEGG enrichment analysis to find possible functions and pathways, and then combined with GO library for GSEA analysis and screening	It involves downloading cell expression data from the GEO database, constructing expression matrices, and performing co-expression analysis of lncRNA and mRNA. GO and KEGG enrichment analyses are conducted to identify relevant functions and pathways, followed by GSEA analysis	High SP100-related pathways were obtained via GO, KEGG and GESA. Among which the lung cancer-related pathways were tumor necrosis factor alpha (TNF-α)-mediated signaling pathway, cysteine end peptidase involved apoptosis signaling pathway, and toll-like receptor signaling pathway.	Upregulation of SP100 gene can interact with apoptotic genes to inhibit the risk of lung cancer associated with cerebral infarction.	High
SP140 inhibits STAT1 signaling, induces IFN-γ in tumor-associated macrophages, and is a predictive biomarker of immunotherapy response [38]	Kiran K, et al.	2022	USA	cohort	SP140 and its role in the signaling of TAMs and response to immunotherapy.	48 HNSCC samples, 21 HNSCC and lung cancer,	Lung cancer, HNSCC and melanoma	head and neck cell lines (HNSCC) and metastatic melanoma	SP140	High expression	Flow cytometry, Cell viability assay, Immunofluorescence and cytokine analysis	We evaluated the correlation between SP140 expression and also used complementary bioinformatics and experimental approaches. Chromatin immunoprecipitation was used to demonstrate the direct binding of SP140 on the promoters of STAT1	SP140 is highly expressed in TAMs across many cancer types, including HNSCCs and was associated with higher tumor mutation burden, improved survival, and a favorable response to immunotherapy.	SP140 could serve as both a therapeutic target and a biomarker to identify immunotherapy responders.	Low
Gene signature based on B cell predicts clinical outcome of radiotherapy and immunotherapy for patients with lung adenocarcinoma [39]	Linzhi Han, et al.	2020	China	Cohort	Presenting a predictive factor for the response of LUAD patients to immune checkpoint inhibitors (ICIs) treatment and RT	423 TCGA-LUAD patients	Lung adenocarcinoma	Lymphocyte B cells	SP110	High expression	308 genes of the 393 upregulated genes in B cells from GEO database were found in TCGA data set. Univariate analysis was used to identify 22 prognosis-related genes, and LASSO COX regression analysis was then used to select 14 independent risk genes. At last, six genes were screened out to construct a B cell‐specific gene signature using multivariate Cox regression analysis.	Kaplan–Meier method, Wilcoxon rank-sum test, Chi-square test, Univariate Cox analysis, Gene Expression Omnibus and The Cancer Genome Atlas (TCGA) databases, Omnibus	Sp110 was an independent prognostic factor.	High expression of SP110 was correlated with poor prognosis.	Low
A genome-wide association study identifies six susceptibility loci for chronic lymphocytic leukemia [40]	Maria Chiara Di Bernardo, et al.	2008	China	bioinformatics and cohort	Identifying susceptibility loci for chronic lymphocytic leukemia	peripheral blood samples were collected from 517 individuals with CLL (364 males, 153 females)	CLL	B-cell)/lymphoblastoid cells	SP140 (LYSp100B)	Polymorphism	Genotyping: Invitrogen, Illumina, Kbiosciences, Mutational status: Chromas software version 2.23 (Applied Biosystems), Statistical analysis: S-Plus 8, R (v2.6) and STATA (v8) software, Bioinformatics: Haploview software (v3.2), MACH1.0 on HapMap,	S-Plus 8, R (v2.6) and STATA (v8) software, x2 test or Fisher’s exact test, Cochran-Armitage trend test.	six previously unreported CLL risk loci at 2q13 (rs17483466),2q37.1 (rs13397985, SP140), 6p25.3 (rs872071, IRF4), 11q24.1 (rs735665), 15q23 (rs7176508) and 19q13.32 (rs11083846, PRKD2)	Polymorphisms of SP140 are associated with risk of CLL.	Low
Genetic susceptibility for chronic lymphocytic leukemia (CLL) among Chinese in Hong Kong [41]	Lan Q, et al.	2010	USA	observational	genome-wide association study (GWAS) of CLL in Hong Kong Chinese for SNPs detection	Seventy-one Chinese patients with CLL, Blood samples obtained anonymously from the residual blood used for clinical testing from 1,273 Chinese patients with non-cancer diagnoses.	CLL	-	SP140	Polymorphism	Genotyping was carried out by TaqMan.	chi-square test, Wilcoxon rank sum test, Fisher’s exact test	SNPs strongly associated with CLL in Caucasians were also associated with CLL in an Asian population.	Polymorphisms of SP140 are associated with risk of CLL.	Moderate
Clinicopathological and molecular characterization of chromophobe hepatocellular carcinoma [42]	Hyo Jeong Kang, et al.	2021	South Korea	Cohort	to understand the clinicopathologic characterization and molecular features of chromophobe HCC	224 cases with curative surgical resection for HCC	Hepatocellular carcinoma	chromophobe hepatocellular tumor cells	SP100	Somatic mutations	Whole exome sequencing, Telomere-specific fluorescence, CIBESORT, KEGG Pathway analysis, HELLMARK set analysis,	whole exome sequencing, copy number variation, and transcriptomic analyses, telomere-specific fluorescence in situ hybridization, SPSS statistical software program (version 18.0 SPSS Inc Chicago, IL, USA), and R (version 4.0.0), χ2 test, Kaplan-Meier method, Wilcoxon rank-sum test, Pearson’s chi-square or Fisher’s exact test, Wilcoxon signed-rank test, log-rank test.	Somatic mutations of SP100 were identified in chromophobe HCC.	chromophobe HCC is associated with female predominance and ALT,	Low
Longitudinal analysis of 25 sequential sample-pairs using a custom multiple myeloma mutation sequencing panel [43]	KM Kortüm, et al.	2015	USA	Cohort	MM-specific targeted sequencing gene panel using for individual tumor characterization, tracking clonal evolution and better guiding personalized treatment strategies	DNA of 22 newly diagnosed and 3 pretreated multiple myeloma patients.	Multiple myeloma	lymphoproliferative cell line (GM19240, Coriell Cell Repositories, Camden, NJ, USA)	SP140	Truncating and missense mutations	whole exome sequencing (WES), single cell analyses	FISH analysis	SP140 is involves in pathogenesis of chronic lymphocytic leukemia, two truncating SP140 mutations (p. Arg576* and p. Glu75*) and one missense mutation (p. Glu856Lys), four DIS3 mutations were identified prior to treatment	However, the clinical impact of mutations in SP140 in MM is not yet determined. However, a 72% decrease in abundance of the missense mutation after treatment happened.	Low
A novel long noncoding RNA SP100-AS1 induces radio resistance of colorectal cancer via sponging miR-622 and stabilizing ATG3 [44]	Zhou, Y, et al.	2023	China	Experimental study	The aim of this study is to investigate the role of a novel long noncoding RNA (lncRNA) called *SP100-AS1* in inducing radio resistance in colorectal cancer. Specifically, the study aims to explore how *SP100-AS1* contributes to this resistance by sponging microRNA-622 (miR-622) and stabilizing ATG3, a gene involved in autophagy.	44 patients, 22 radiosensitive and 22 radioresistant	colorectal cancer	Colorectal cancer cell lines HCT116, SW480, LS174T, CT26, HT29, and LoVo.	SP100	Different changes	qPCR and Western Blot	To investigate the differences in radiosensitivity between CRC patients and cell lines, focusing on RNA sequencing, cell culture, animal models, and various assays for functional and molecular analysis.	1-SP100-AS1 upregulation is associated with CRC radio resistance 2-SP100-AS1 knockdown enhances CRC radiosensitivity in vitro 3-SP100-AS1 confers significant radio resistance in vivo	SP100-AS1 silencing could enhance the radiosensitivity of CRC in vitro and in vivo. The present study indicates that SP100-AS1/miR-622/ATG3 axis contributes to radio resistance and autophagic activity in CRC patients, suggesting it has huge prospects as a therapeutic target for improving CRC response to radiation therapy.	Low
SP110 Could be Used as a Potential Predictive and Therapeutic Biomarker for Oral Cancer [13]	Xu, G. Q, et al.	2024	China	Experimental study	The study aims to investigate the prognostic significance of SP110 in oral cancer.	27 oral cancer patients	Oral cancer	SCC-9 CAL-33 and SCC-25	SP110	High expression	Immunohistochemistry	The study uses bioinformatics and lab experiments to analyze SP110 expression in OSCC tissues, assessing it as a potential biomarker and therapeutic target.	The study revealed that high SP110 expression is linked to poor prognosis in oral squamous cell carcinoma (OSCC), suggesting it could be a predictive biomarker for OSCC progression and a potential therapeutic intervention target.	High SP110 expression is linked to poor prognosis of oral cancer.	High
Unraveling neoantigen-associated genes in bladder cancer: An in-depth analysis employing 101 machine learning algorithms [45]	Fang Lv, et al.	2024	China	In silico bioinformatics analysis (Retrospective cohort study using public databases)	Insights into the role of neoantigens in BLCA	• TCGA-BLCA cohort (*n* = 397) • GSE13507 (*n* = 161) • GSE32894 (*n* = 224) • IMvigor210 immunotherapy cohort (*n* = 298) • GSE176307 immunotherapy cohort (*n* = 89) • GSE129845 single-cell data (3 patients)	Bladder cancer (BLCA)	T cells, B cells, myeloid cells, fibroblasts, epithelial cells, endothelial cells, and smooth muscle cell groups	SP140	NA	RNA sequencing data analysis, machine learning algorithm (Random Survival Forest + Elastic Net), immunohistochemical validation (from HPA database, though for other genes).	SP140 was one of nine genes used to construct a prognostic risk score model using the RSF + Enet[alpha = 0.9] machine learning algorithm.	SP140 was noted to exhibit a higher correlation with drug sensitivity compared to other model genes like ID2 and PTMS. SP140 had a negative coefficient in the risk model, meaning higher expression might be protective.	The study concludes that the 9-gene prognostic model (including SP140) is a robust tool for prognosis prediction in BLCA. It suggests the model can guide frontline clinicians in diagnosis, treatment, and prognosis prediction. The specific biological mechanism of SP140 in BLCA was stated as unreported.	High

### Findings

#### SP100 expression and alterations

SP100 expression was reported in 11 studies across cancers, including HCC, lung cancer, breast cancer, PAAD, glioma, mantle cell lymphoma, laryngeal cancer, CRC, and ccRCC. High SP100 expression was observed in breast cancer, lung cancer, glioma, and PAAD. In glioma, PAAD ‘s high expression was correlated with poor prognosis, while in breast cancer and lung cancer, it was associated with better outcomes or reduced invasiveness. Conversely, low SP100 expression was noted in LSCC and linked to tumorigenesis and poor differentiation. In CRC, it was shown that SP100-AS1 axis silencing could enhance the radiosensitivity, while its overexpression was associated with CRC radio resistance. Genetic alterations included polymorphisms in MCL and somatic mutations in chromophobe HCC. Epigenetic changes were less frequently reported for SP100, though its expression was modulated by interferon-alpha in breast cancer.

#### SP110 expression

SP110 was evaluated in 7 studies involving various cancers: DLBCL, PAAD, oral cancer, lung adenocarcinoma, glioma, and ccRCC. High SP110 expression was consistently associated with poor prognosis in oral cancer, lung adenocarcinoma, PAAD, and ccRCC. Its low expression was noted in DLBCL, suggesting a protective role. In PAAD, high expression of SP110 was correlated with a better prognosis, while in lung adenocarcinoma and OSCC, it was associated with a poor prognosis.

#### SP140 expression

SP140 was the most frequently studied family member, appearing in 17 studies across several cancers. Expression patterns were highly variable: high SP140 expression was linked to poor prognosis in ccRCC, glioma, LSCC, ovarian and cervical cancers but associated with better prognosis in AML, osteosarcoma, HNSCC, lung cancer, and metastatic melanoma. Low SP140 expression was reported in MCL and correlated with poor progression-free survival. Genetic alterations included mutations (e.g., frameshift/nonsense in MCL, truncating/missense in MM), translocations (e.g., with PD-L1 in DLBCL). Copy number loss of SP140 in CSCC was correlated with poor prognosis. Epigenetic changes, such as hypomethylation in AML and hypermethylation in CMML, were also noted, often tied to TET2 mutations. In AML, hypomethylation of SP140 was correlated with curable AML and a better prognosis. Confirmed by two independent studies, polymorphisms of SP140 were associated with the development of CLL. Mechanistically, studies suggest that SP140 promotes PD-L1 expression through STAT1/IRF1 activation in tumor-associated macrophages, which may explain its association with immune escape and poor prognosis in cancers such as glioma and ccRCC. In BLCA, SP140 was noted to exhibit a higher correlation with drug sensitivity compared to other model genes like ID2 and PTMS [45].

#### SP140L expression

SP140L was the least studied member. It was reported and associated with a poor prognosis in PAAD, with deep deletions and missense mutations identified in glioma.

## Discussion

This systematic review synthesizes evidence from 29 studies spanning 2004 to 2024, elucidating the expression patterns, genetic/epigenetic alterations, prognostic implications, and mechanistic roles of SP100 family proteins across 25 cancer types. The findings reveal a complex, cancer-dependent role for these proteins, with significant variability in expression and clinical outcomes across hematologic malignancies and solid tumors.

### Hematologic malignancies

Hematologic cancers, including AML, CMML, MCL, DLBCL, CLL, and MM, were investigated, reflecting the SP100 family’s prominence in these malignancies. In AML, SP140 hypomethylation was consistently associated with better prognosis and curable disease. DNA methylation is one of the epigenetic processes that regulates gene expression in stem cell physiology, normal differentiation, and the development of cancer [46]. Several genes encoding DNA methylation enzymes, including TET2 (ten-eleven translocation (TET) oncogene family member 2), which is a tumor suppressor, are mutated in AML. Hypomethylation of SP140 improves AML outcomes due to its interaction with TET2-mediated DNA methylation pathways [23]. This contrasts with CMML, where SP140 hypermethylation in TET2-mutant patients correlated with disease progression and proposed a good marker for identifying TET2-mutant patients [47]. These epigenetic differences highlight SP140’s dual role, modulated by methylation status, and highlight its potential as a prognostic marker in myeloid leukemias.

In MCL, SP140 downregulation via mutations (8% frameshift/nonsense and 7.5% deletions) was linked to poor progression-free survival. Mechanistically, SP140 mutations caused downregulation of BCR signaling and MYC targets, leading to cancer progression and poor prognosis [27]. While SP100 polymorphisms were implicated in MCL pathogenesis, SP100’s association with TP53 may contribute to genomic instability and cancer development [28].

CLL studies identified SP140 polymorphisms as risk factors, with mechanistic links to immune dysregulation, though direct expression data were limited [40, 41]. In MM, SP140 mutations (truncating/missense) were detected but lacked clear clinical impact, suggesting a gap for further studies [43]. SP110 being downregulated in DLBCL, by disrupting natural killer cells’ performance, was suggested as a marker to predict prognosis and treatment efficacy [33]. On the other hand, translocation of SP140, by inducing PDL-1 overexpression, was associated with DLBCL development [32]. This can suggest the SP140 gene as a new therapeutic target for DLBCL.

Collectively, these hematologic malignancies illustrate the SP100 family’s involvement in immune modulation and genomic stability, with SP140 emerging as a key player. These findings can be the foundation of future studies regarding the SP100 family as new targets in the treatment of hematologic malignancies.

### Solid tumors and non-hematologic malignancies

In glioma, all four members of the SP100 family were overly expressed, which was correlated with poor prognosis. SP140 was further announced as a practical tool for predicting the survival of glioma patients and also as an effective treatment target. SP140 inhibitor can suppress glioma progression via TRIM22/PI3K/AKT signaling pathway [14, 31]. The SP140 is an epigenetic reader protein associated with nuclear response that is overexpressed in immune cells. It is a novel determinant of transcriptional repression due to its inclusion of a SAND domain—a DNA-binding domain present in other immune regulators, AIRE and DEAF1. Thus, SP140 can bind specifically to regions of silenced chromatin. SP140 is recruited to regions of chromatin marked by histone H3 lysine 27 trimethylation (H3K27me3), where it binds and recruits PRC2 and NuRD repressive complexes to silence lineage-inappropriate or proinflammatory genes. Such transcriptional repression is critical for sustained identity of immune cell phenotype and prevents unyielding activation of genes [48, 49]. Interestingly, in the glioma and clear cell renal cell carcinoma (ccRCC) tumor microenvironments, expression of SP140 is dysregulated, resulting in altered macrophage polarization and immune evasion. SP140 is also an epigenetic reader that skews inflammation-related transcriptional programs in tumor-associated macrophages (TAMs). SP140 directly binds to the promoter and alters chromatin, which affects downstream transcriptional regulators such as STAT1 and IRF1, which get upregulated with IFN-γ signaling that’s known to promote PD-L1 expression. SP140-high tumors possess increased PD-L1 transcription levels as SP140 acts to facilitate an immunosuppressive tumor microenvironment. Such upregulation of PD-L1 inhibits the activity of cytotoxic T cells and decreases the responsiveness of tumors to PD-1/PD-L1 immune checkpoint inhibitors. Thus, SP140-high tumors are inherently or adaptively resistant to immunotherapy, and resistance assessment should include SP140-mediated epigenetic regulation [38, 50, 51]. For example, SP140 silences genes related to innate immunity but overlaps with upregulation of pattern recognition receptors (PRRs) RAGE and TLR4. Both RAGE and TLR4 are upregulated in tumor-associated macrophages (TAMs) and serve pro-inflammatory purposes for sustained inflammation, angiogenesis, and tumor progression [52–54]. Thus, when SP140 is dysregulated, its powers of gene silencing on immune-relevant genes can be co-opted for tumor-promoting inflammatory signaling instead. LSCC studies reported high SP140 and low SP100 expression, both linked to aggressive disease. SP140’s overexpression may enhance immune responses in the tumor microenvironment, while SP100’s downregulation disrupts differentiation via transcriptional/translational suppression, leading to initiation and progression of tumorigenesis [10, 25].

In examining the role of SP140 in 225 patients with osteosarcoma, this protein, in addition to having a protective role, by being overexpressed promises a better prognosis [29, 30]. Furthermore, breast cancer exhibited high SP100 expression with favorable outcomes, mediated by ETS1 repression and IFN-α modulation, highlighting a tumor-suppressive role for SP100 distinct from other cancers [11, 36]. Lung cancer mirrored this, with high SP100 and SP140 expression linked to better prognosis, involving apoptotic (TNF-α, caspase) and TAM-mediated immune pathways, respectively [38, 39, 55]. By activating STAT1/IRF1 signaling through IFN-γ in tumor-associated macrophages, SP140 promotes PD-L1 expression, which has been reported as both a therapeutic target and a predictive biomarker of immunotherapy response in cancers such as metastatic melanoma and head and neck neoplasms [39].

In BLCA, the mentioned finding that SP140 expression correlates highly with drug sensitivity profiles makes it a compelling candidate for further research. It could be developed into a companion diagnostic test to determine if a patient is a good candidate for a specific targeted therapy [45].

In PAAD, high expression of SP100, SP110, SP140, and SP140L consistently correlated with poor prognosis (Yunjie Duan, 2023), in contrast with another study linking SP110 to better outcomes. One hypothesis for this matter is SP110’s prognostic impact depends on the immune context of the PAAD tumor microenvironment (TME). Zhu L, et al. emphasize immune cell infiltration, noting differential expression of seven immune cell types (e.g., Th17 cells) in high- versus low-risk groups, with SP110 upregulation potentially enhancing anti-tumor immunity (via NF-κB or Th17-mediated responses). In contrast, Yunjie Duan (2023) reports lower SP110 expression in tumor-infiltrating immune cells, suggesting immune suppression that favors tumor growth. These different immune environments can affect the results. On the other hand, variations in sample size, data sources, and analytical approaches can account for the conflicting results [16, 34]. As an evolutionary relative of SP140, SP140L in primate subpopulations also has domains of functionality such as SAND domain for DNA binding, PHD for methyl-histone reading, and BRD for acetyl-lysine binding, which would similarly imply chromatin-related immune regulatory features. While no studies exist directly in PAAD, SP140L’s ability to downregulate interferon-stimulated genes and viral DNA sensing in B cells reveals the possibility of downregulating tumor-associated macrophage phenotypic reprogramming and immune checkpoint expression. Thus, we predict that SP140L may regulate PD-L1 or RAGE/TLR4 in PAAD through epigenetic silencing of inflammatory loci, which subsequently promotes immune escape. This would be comparable to SP140’s upregulatory effects on PD-L1 via STAT1/IRF1 activation in other tumors [56]. Recently, SP100A expression was validated as a novel regulatory target of N¹-methyladenosine (m¹A) RNA modification post-transcriptional regulation via activity of demethylase ALKBH3. This ALKBH3–SP100A regulatory relationship represents a novel post-transcriptional epitranscriptomic layer influencing SP100A’s functional output, complementing traditional genomic and transcriptional regulatory mechanisms. ALKBH3-dependent m¹A demethylation promotes SP100A mRNA stability and translational efficiency to yield SP100A protein levels and subsequent tumor suppressive functions down the line of this factor. Thus, this type of regulation dictates tumor-type-specific ranges of SP100A; such fluctuations may explain the differing prognostic meaning of SP100A seen in glioma, melanoma, and ccRCC. Moreover, SP100A is a common biomarker across many tumor types with confounding features; as such, it is subject to post-translational regulation like RNA methylation, alternative splicing, and miRNA-mediated decay, which can lower protein availability without impacting mRNA expression levels. Thus, studies believe SP100A was functionally active based on transcriptome studies alone and could have been led astray. To reconcile the discrepancies, future investigations should include proteomic and epitranscriptomic evaluations wherein conclusions make more sense with assessments of other regulatory levels beyond transcription [57, 58].

Ultimately, the TME is a complex immune ecosystem of immune cells, stroma, cytokines, and the extracellular matrix that ultimately determines how responsive the tumor is to immunotherapy. For example, in gastric cancer, the overall number of suppressive immune cells and their location in the tumor microenvironment—M2 polarized macrophages, Tregs, and MDSCs—compromise the efficacy of PD-1/PD-L1 blockade. These suppressive immune cells are accompanied by immunosuppressive cytokines—IL-10, TGF-β, and VEGF—that decrease cytotoxic T cell activity and upregulate PD-L1 on TILs and other immune cells, respectively. Rare but impactful papers demonstrate the influence of the inflammatory microenvironment and these regulatory molecules on response to sintilimab anti-PD-1 immunotherapy. For instance, high expression of PLIN3 and EPHB2 is associated with macrophage infiltration and poor response to immunotherapy in gastric carcinoma and other solid tumors [59, 60]. High expression of hypoxia-related gene signatures also correlates with immune exclusion and resistance to immune checkpoint inhibitors [61].

SP Family proteins represent important novel findings related to immune gene accessibility and expression. SP140 and SP100 can influence gene programs in macrophages and dendritic cells; thus, they would also likely influence PD-L1 expression as well as pro-inflammatory signaling in the tumor microenvironment. Thus, pharmacologic or genetic targeting of SP family proteins would rebalance the immune landscape in gastric cancers by reducing SP-associated immunosuppressive infiltrating cells and shifting toward an activated T cell phenotype—resulting in increased sensitivity to immunotherapy [38, 50, 51].

**Fig. 2:**
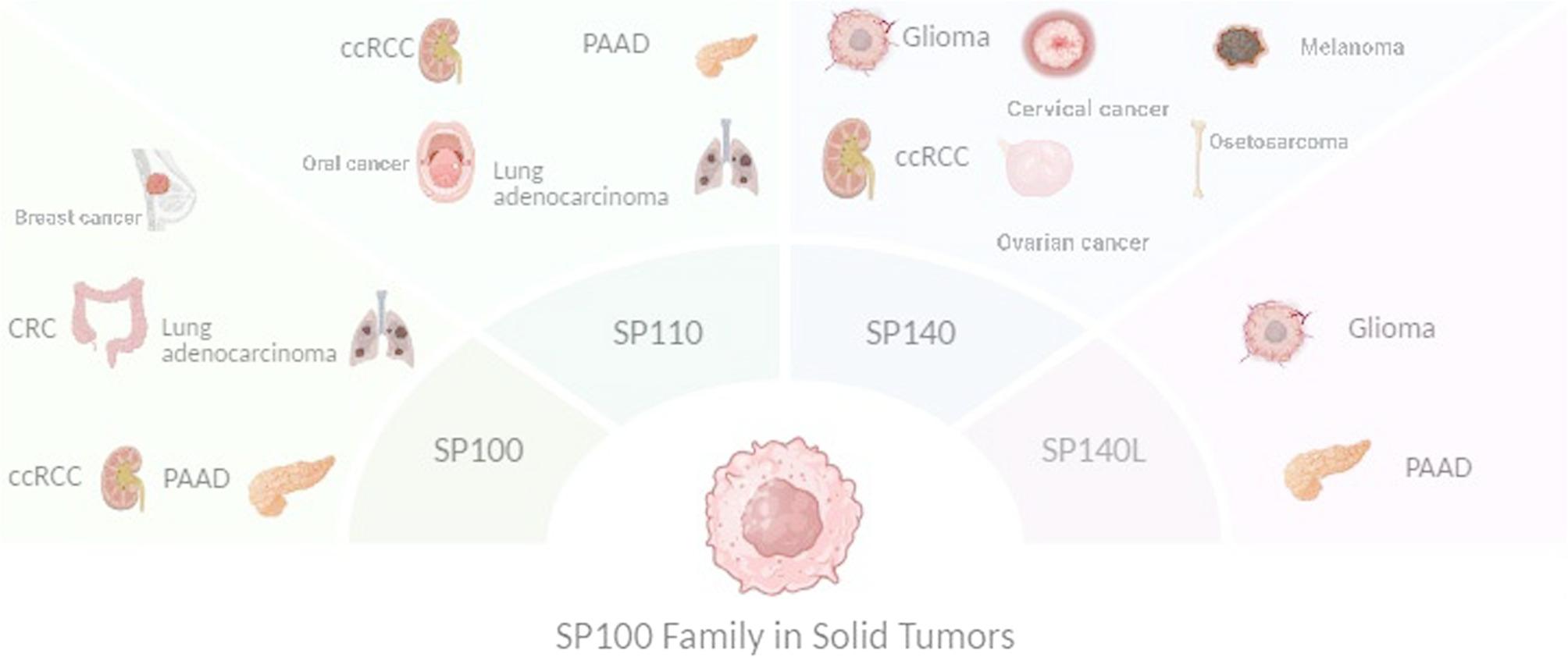
Distribution of SP100 family proteins across various solid tumors. Graphical representation showing the association of each SP100 family member (SP100, SP110, SP140, and SP140L) with different solid tumor types, as identified in the reviewed literature. The diagram summarizes reported cancer types for each protein, highlighting their distinct and overlapping tumor associations. Abbreviations: CRC – colorectal cancer; ccRCC – clear cell renal cell carcinoma; PAAD – pancreatic adenocarcinoma

The SP100 family’s clinical significance hinges on its expression context and cancer-specific mechanisms, while mechanistically, SP100 family proteins regulate cancer via immune modulation, epigenetic alterations, and oncogenic signaling. SP100 is a subunit of promyelocytic leukemia nuclear bodies (PML-NBs) and binds to heterochromatin protein 1 (HP1), contributing to transcriptional repression and chromosomal architecture. By strengthening HP1’s binding to PML-NBs, increasing heterochromatin formation, and silencing oncogenic loci, this association contributes to SP100’s antitumor effects [62]. Ultimately, when this axis is perturbed, genomic instability occurs, which results in loss of tumor suppressive function. In a surprising twist, however, herpes simplex virus type 1 (HSV-1) usurps SP100 for its replicative needs. The immediate-early protein ICP0 functions as an E3 ubiquitin ligase to target SP100 for proteasomal degradation, which disrupts PML-NBs and facilitates ICP0 function against host antiviral response [63]. This is similarly observed in a variety of malignancies where SP100 is degraded and loss of PML-NB integrity is associated with tumor progression and immune evasion [64]. Thus, the results suggest that SP100 serves as both a viral restriction factor, and a tumor immune factor which can be manipulated for therapeutic purposes. Their nuclear body localization (e.g., PML-NBs) and interactions with TP53 mutations, as well as DNA repair pathways, further amplify their impact. These insights suggest SP140 inhibitors (e.g., GSK761 in glioma) and SP100-AS1 silencing (in CRC) as promising strategies, while SP100’s IFN-α responsiveness in breast cancer could enhance immunotherapy. Translational cancer applications that block the S100/RAGE axis have emerged in the oncology literature. For example, in pancreatic ductal adenocarcinoma (PDAC), an intraperitoneal injection of a SP100 derived RAGE antagonist peptide (RAP) inhibited tumor growth and metastasis via attenuation of ligand-dependent activation of NF-kB [65]. In a model of smoke-induced lung carcinogenesis, silencing S100A8/A9 with siRNA or silencing RAGE resulted in decreased tumor growth and proinflammatory signaling, with findings suggesting that this pathway sensitizes immune responses to facilitate tumor growth [66]. Therefore, the potential for translation of RAGE blockade is warranted, even in malignancies that do not have a largely reported SP family proteomics association. GSK761 has been explored as a selective SP140 inhibitor with potential immunomodulatory effects. However, with studies planned for translation, incremental adjustments should be made with immune checkpoint inhibitors and planned assessments of highly inflamed tumors for best results. A graphical summary of the distribution of SP100 family proteins in different solid tumors is provided in Fig. 2.

### Mechanistic insights into the SP100 family: pathways in Inflammation, chromatin remodeling, and immune modulation

The SP100 family (SP100, SP110, SP140, SP140L)—functions as decoy nuclear chromatin readers who are involved in immune signaling apart from transcriptional repression through different downstream pathways. Where SP100 is situated in an offensive position, it bioenergetically engages with PML and HP1 as a nuclear body, regulating chromatin compaction and transcriptionally repressing pro-oncogenic gene expression; thus, the diminished presence of SP100 upregulates pro-oncogenic NF-kB, RAS, and MYC superpathways, all of which facilitate transformation to cancer [5]. SP110 acts offensively as a leukocyte expression that mediates SP110 with macrophages to downregulate inflammation gene expression via altered TLR4 activity or STAT1 mediation during myeloid differentiation; therefore, mutations in immune SP110 are associated with immunodeficiency syndromes, and SP140 acts as a chromatin reader that enhances STAT1/IRF1 activity and promotes immune checkpoint evasion. It correlates with increased PD-L1 expression in macrophages, thereby dampening anti-tumor immunity and facilitating tumor progression [38]. Meanwhile, the most poorly studied, SP140L, contains a conserved bromodomain across species and a SAND domain, and multiple other domains suggesting epigenetic reprogramming during EMT when responding to an interferon-rich microenvironment [4, 56]. Targeting SP140 in this paper through silencing in the glioma and ccRCC murine models or GSK761 inhibition provided sentinels for ease of association/reduction, as TRIM22 as well as PI3K/AKT and TLR4-related PD-L1 levels were reduced post-treatment, resulting in reduced tumor invasiveness and re-sensitized T cells [54, 67]. Thus, the SP family has varied roles both in epigenetic regulation and immune evasion, providing a mechanistic rationale from which to expand therapeutic efforts and bioinformatic candidates for glioblastoma, ccRCC, and gastric carcinoma.

### Mechanistic roles of SP100 family proteins in key cancer-related processes

#### SP100 and cell growth: the STAT1 connection

SP100 regulates a protein called STAT1 to control cell division. Usually, STAT1 suppresses cell division, but whenever necessary, it can also eliminate cells. Yet as SP100 levels decrease, STAT1 is suppressed, which causes uncontrolled division of cells and may result in traits like cancer. By stabilizing the cells, restoring SP100 levels strengthens STAT1’s ability to prevent cell division. Since the action of STAT1 varies based on the type of cancer, SP100 tends to guide it toward its growth-limiting role [12–69].

#### SP140 and immune escape: the PD-L1 link

The tumor microenvironment is formed by SP140, a chromatin reader that is primarily expressed in immune cells and controls immunological checkpoints. Based on recent studies, SP140 enhances STAT1 signaling in tumor-associated macrophages, which subsequently increases the expression of PD-L1. By elevating PD-L1 levels, SP140 promotes immune evasion, reduces T cell activity, and contributes to tumor progression. Accordingly, SP140 serves as a biomarker for predicting immunotherapy response, sometimes showing even higher predictive value than PD-L1 itself [70, 71].

#### SP110’s role in myeloid cell differentiation

SP110 is a member of the nuclear body protein family SP100/SP140. It is very important in regulating the development of myeloid cells, which are the body’s first line of defense. Leukocytes are where it is mostly found. It acts as a transcriptional coactivator, helping to turn on genes that instruct immature immune cells what direction to take. SP110 seems to be more than just a passive player; it actively changes the cellular environment that is important for healthy differentiation since it has an impact on ribosome biogenesis and nuclear hormone receptor signaling. It’s remarkable how alternative splicing has led to several protein isoforms, and each one may be able to benefit different parts of the development of immune cells. When SP110 is disrupted, it can lead to serious immune deficiencies, highlighting just how vital it is to the immune system’s balance and function [72].

#### SP140L in chromatin regulation and EMT

The newest member of the SP100 protein family, SP140L, changes how accessible chromatin is and may be affecting the epithelial-mesenchymal transition (EMT), which is important for cancer development. SP140L may detect changes in histones because it has conserved domains like the bromodomain, plant homeodomain (PHD), and SAND domain [4]. Researchers have found that SP140L is in the same nuclear bodies as SP100 and SP140, which suggests that they all have a similar function of repressing transcription and remodeling chromatin. However, SP140L has not been studied as much as its relatives. New research suggests that SP140L may affect EMT by changing how easily genes involved in cell adhesion, migration, and immunological signaling can be accessed, especially in settings with a lot of inflammation or interferon. Its ability to be induced by interferon and only expressed in B cells and monocytes further supports its possible role in changing the tumor microenvironment and controlling immune-driven epigenetic alterations. SP140L’s chromatin-binding elements and immune-specific activities make it a promising candidate for more studies on cancer development and metastasis, even though connections to EMT are still being explored [56].

**Fig. 3:**
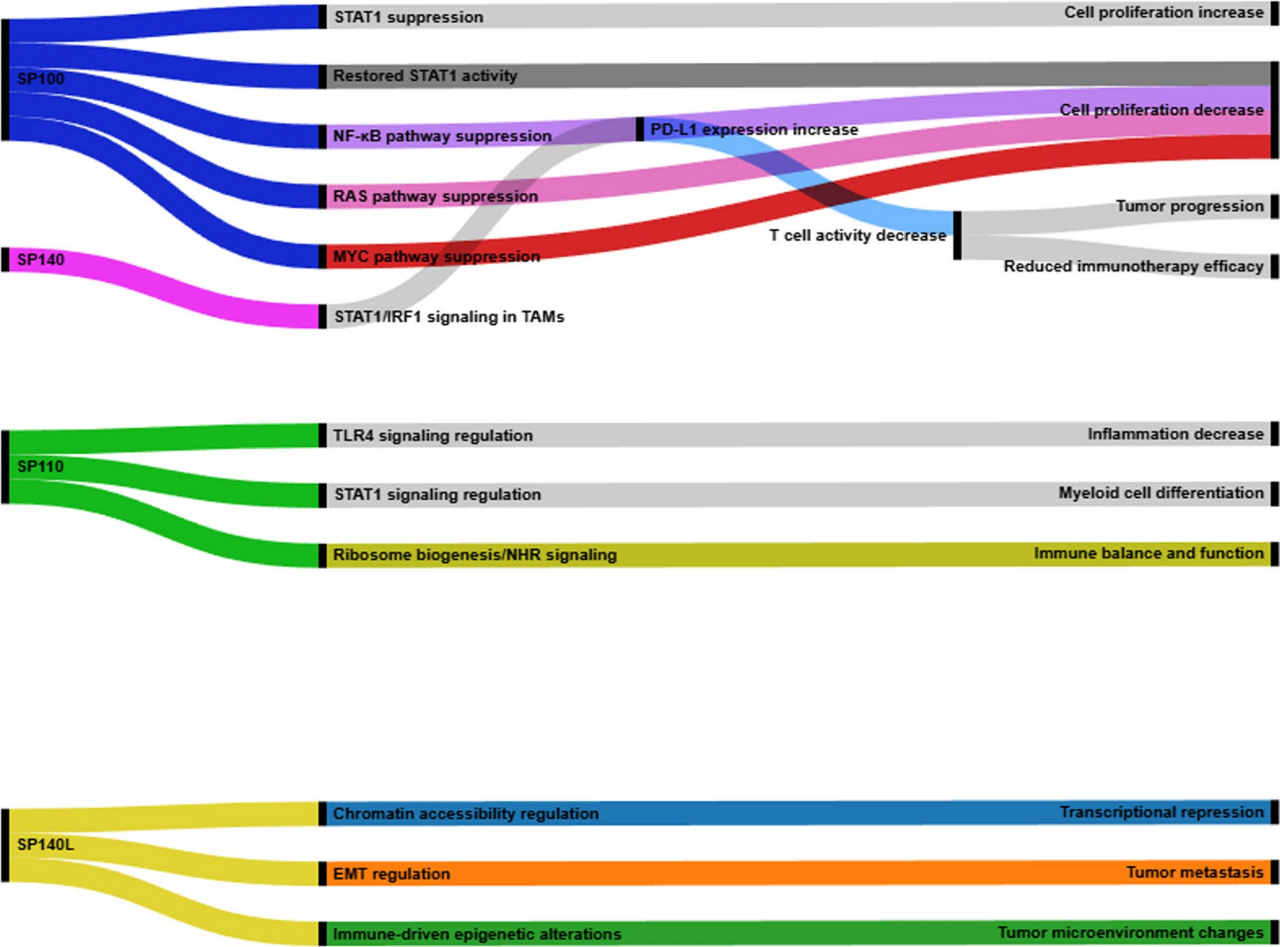
Mechanistic Roles of the SP100 Protein Family in Cancer Pathways. Mechanistic roles of SP100 family proteins in cancer progression and immune regulation. The Sankey diagram illustrates the distinct yet interconnected pathways through which SP140, SP100, SP110, and SP140L influence tumor biology. SP140 enhances STAT1/IRF1 signaling in tumor-associated macrophages, increasing PD-L1 expression, suppressing T-cell activity, and reducing immunotherapy efficacy. SP100 suppresses NF-κB, RAS, and MYC pathways to curb cell proliferation. SP100 also regulates STAT1, where its suppression promotes uncontrolled proliferation, while restoration of SP100 strengthens STAT1’s growth-limiting activity. SP110 regulates TLR4 and STAT1 signaling, decreasing inflammation and directing myeloid cell differentiation. SP140L alters chromatin accessibility and contributes to EMT regulation, contributing to metastasis. These mechanistic links highlight the SP100 protein family’s pivotal roles in tumor suppression, immune modulation, and therapeutic responsiveness

A summary of these mechanistic interactions across the SP100 protein family (SP100, SP110, SP140, SP140L) is presented in Fig. 3, highlighting their shared and distinct roles in tumor progression and immune regulation.

### Clinical translation potential and challenges

The SP family has been recognized in therapeutic studies of late, specifically SP140, as a viable immunotherapy and epigenetic target. As such, a small molecule inhibitor was created, GSK761, as a selective SP140 inhibitor. GSK761 was shown to successfully modulate macrophage polarization via decreased chromatin binding of SP140 and reduced levels of proinflammatory cytokines, in which SP140 has been recognized as a druggable epigenetic target in immune-related and inflammatory cancers [73]. In vivo studies within murine glioma models show that GSK761 inhibits glioma progression via TRIM22 downregulation and downregulation of the PI3K/AKT pathway, which suggests that SP140 inhibition may allow for reduced glioma invasiveness [74]. Compounding such findings were studies that assessed activators/inhibitors of SP140/TLR4 signaling in glioblastoma and clear cell renal cell carcinoma (ccRCC), which showed that when TLR4 is activated, PD-L1 levels are upregulated, allowing for immune evasion and immune escape from checkpoint blockade [67]. Complementary studies show that tumors with an SP profile dynamically regulate PD-L1, and where SP140 is present, PD-L1 levels on tumor-associated macrophages facilitate immunosuppression and predict responses to anti-PD-1/PD-L1 therapies [38]. Thus, modulation of the SP-140 profile may be translatable to predict response to anti-PD-1/PD-L1 immunotherapies and predict response to personalized treatment regimens, even circumventing immune-related adverse events. However, while these preclinical findings are promising, clinical translation requires validation through large-scale, controlled trials to evaluate efficacy, safety, and optimal therapeutic combinations.

### Limitations and future directions

This review’s findings are tempered by heterogeneity in study designs, sample sizes, and risk of bias (37.9% moderate, 10.3% high). The predominance of Chinese studies (76%) may introduce geographic bias, and SP140L’s sparse data restricts its evaluation. Future research should prioritize multi-center studies, standardize expression measurement (e.g., qPCR, RNA-seq), and explore SP140L’s role. Mechanistic studies targeting SP140’s immune pathways (e.g., PD-L1, TRIM22) and SP100’s DNA repair functions in underrepresented cancers (e.g., BLCA, OSCC) could refine therapeutic applications. This study strongly recommends further research on the alteration of SP110 in PAAD, as it can be used as a prognostic marker and treatment target alongside other SP100 family members. Longitudinal cohort studies and clinical trials testing SP100 family modulators are also critical to translate these findings into practice.

## Conclusion

This systematic review underscores the dual oncogenic and tumor-suppressive roles of SP100 family proteins (SP100, SP110, SP140, SP140L) across 25 cancer types, shaped by context-dependent expression patterns, genetic/epigenetic alterations, and possible interactions with tumor microenvironments. This study revealed several roles for members of this family, including that SP140 is a pivotal prognostic biomarker in hematologic malignancies (e.g., hypomethylation in AML predicts favorable outcomes, while mutations in MCL drive aggressive disease). In solid tumors, this review uncovers novel tissue-specific roles: SP140 promotes glioma progression but protects osteosarcoma, while SP100 suppresses breast and lung cancers yet exacerbates PAAD. By integrating fragmented evidence, this review provides a framework for leveraging SP100 family proteins as biomarkers and therapeutic targets, bridging molecular insights into clinical translation. It prioritizes future research on SP110/SP140L in immune-microenvironment crosstalk and urges clinical validation of SP100-targeted therapies to advance precision oncology.

As new research around nuclear proteins continues, the importance of the SP100 family, especially SP140, in cancer immunity and epigenetic regulation is growing. Due to SP140’s ability to enhance STAT1/IRF1 signaling and increase PD-L1 expression in tumor-associated macrophages, it has emerged as a crucial biomarker of immune evasion and predictor of immunotherapy resistance in cancers such as HNSCC and lung cancer. Furthermore, not only do they, the SP family, have a crucial role in immunomodulation, but the family’s conserved chromatin-reading domains make them interesting targets for epigenetic drug development, more so as the multi-omics technologies continue to show influence on transcriptional silencing, histone recognition, and inflammation-driven genes. With the knowledge we have today, SP100 and SP140 can be considered potential candidates for further exploration in precision oncology, especially for complex malignancies where chromatin remodeling and immune evasion may lead to therapeutic resistance, such as clear cell renal cell carcinoma (ccRCC) and gastric cancer. Because they influence both immune regulation and epigenetic mechanisms, these proteins represent a potential avenue for developing therapies that could target multiple pathways, pending further validation. If validated in prospective studies, these proteins could contribute to future strategies in personalized cancer care.

## Supplementary Information

Supplementary Material 1.

## Data Availability

Data is provided in the manuscript.
